# Self-Care Behaviors and Dietary Habits Among Patients with Heart Failure: A Cross-Sectional Study

**DOI:** 10.3390/nu18172745

**Published:** 2026-08-22

**Authors:** Anastasia A. Chatziefstratiou, Konstantinos Giakoumidakis, Nikolaos V. Fotos, Andreas Christofi, Sofia Asimina Gavathoglou, Evridiki Patelarou, Hero Brokalaki

**Affiliations:** 1 Cardiac Surgery Unit, General Pediatric Hospital of Athens “Agia Sophia”, 11527 Athens, Greece; a.chatziefstratiou@yahoo.gr; 2 Laboratory for the Development of Self-Care Skills in Patients with Chronic Diseases, Department of Nursing, School of Health Sciences, National and Kapodistrian University of Athens, 11527 Athens, Greece; nikfotos@nurs.uoa.gr (N.V.F.); heropan@nurs.uoa.gr (H.B.); 3 Department of Nursing, School of Health Sciences, Hellenic Mediterranean University, 71410 Heraklion, Greece; epatelarou@hmu.gr; 4 Laboratory of Evidence-Based Healthcare, Education and Clinical Protocols, Department of Nursing, School of Health Sciences, Hellenic Mediterranean University, 71410 Heraklion, Greece; 5 Department of Nursing, School of Health Sciences, National and Kapodistrian University of Athens, 11527 Athens, Greece; andreaschristofi7@gmail.com (A.C.); sofia_gavath@hotmail.com (S.A.G.)

**Keywords:** heart failure, self-care, dietary habits, nutrition, chronic disease management

## Abstract

Background: Self-care and healthy dietary habits are fundamental components of heart failure (HF) management, yet the relationship between dietary quality and self-care remains insufficiently explored. This study investigated the association between dietary habits and self-care behaviors among patients with HF. Methods: A cross-sectional study was conducted among 137 adults with HF attending outpatient clinics in Greece. Self-care behaviors were assessed using the Hippocratic Heart Failure Self-Care Scale (HHFSCS), and dietary quality was evaluated using the validated Greek version of the Cardiovascular Diet Questionnaire-2 (CDQ-2). Associations between demographic characteristics, dietary measures, and self-care were examined using non-parametric tests and multivariable general linear models adjusted for demographic characteristics. Results: The mean HHFSCS score was 41.0 ± 11.3, indicating a moderate level of self-care. Higher scores were observed for medication adherence and appointment keeping, whereas lower scores were observed for exercise, smoking-related behaviors, alcohol-related behaviors, and symptom monitoring. The mean total CDQ-2 score was 8.57 ± 7.17. Overall dietary quality was not significantly associated with total self-care after adjustment for age, sex, marital status, educational level, and employment status (B = −0.102, 95% CI: −0.338 to 0.134, *p* = 0.394). Among the dietary components, only the saturated fatty acid (SFA) score remained significantly associated with total self-care after adjustment (*p* = 0.015), whereas fruit and vegetable and monounsaturated fatty acid scores were not significant. Employment status was also significantly associated with self-care (*p* < 0.001). Conclusions: Overall dietary quality was not significantly associated with total self-care in this sample of patients with HF. However, the observed association between the SFA component and self-care suggests that specific aspects of dietary behavior may warrant further investigation. Given the cross-sectional design and reliance on self-reported measures, these findings should be interpreted cautiously and do not establish directionality or causality. Prospective studies incorporating objective dietary and behavioral measures are needed to further clarify the relationship between dietary habits and self-care in HF.

## 1. Introduction

Cardiovascular disease is the leading cause of death among non-communicable diseases worldwide, according to the World Health Organization (WHO). Cardiovascular disease is defined as a group of disorders affecting the heart and blood vessels, including coronary artery disease (stable angina and acute coronary syndrome) and stroke. Cardiovascular disease also encompasses heart failure (HF) [1]. HF is a major cause of morbidity within the spectrum of cardiovascular diseases. Globally, approximately 1–2% of the adult population is affected by HF, with prevalence increasing to 10% among individuals older than 70 years [1]. In the United States, an estimated 6.7 million adults aged over 20 years are living with heart failure, and this number is projected to increase to 8.7 million by 2030, 10.3 million by 2040, and 11.4 million by 2050 [2]. In Europe, the prevalence of heart failure is estimated at 17 cases per 1000 inhabitants, ranging from fewer than 12 cases per 1000 inhabitants in Greece and Spain to more than 30 cases per 1000 inhabitants in Lithuania [3].

HF is characterized by symptoms such as dyspnea, fatigue, peripheral edema, and reduced exercise capacity, which may substantially affect patients’ functional status and daily activities. The New York Heart Association (NYHA) functional classification categorizes patients into four classes according to the degree of limitation imposed by symptoms. Class I indicates no limitation of ordinary physical activity, Class II slight limitation, Class III marked limitation, and Class IV symptoms at rest and the inability to undertake physical activity without discomfort [4]. The management of HF is based on both pharmacological and non-pharmacological interventions, aiming to alleviate symptoms and slow disease progression. The success of treatment depends not only on the individualized application of therapeutic strategies but also on the adoption of appropriate self-care behaviors [4].

Self-care is based on a broad range of activities that extend from lifestyle practices to the management of health-related problems. Various definitions of self-care have been proposed. The WHO defines self-care as “the activities undertaken by individuals, families, and communities with the intention of enhancing health, preventing disease, limiting illness, and restoring health” [5].

Self-care in patients with HF includes adherence to prescribed medication, symptom monitoring, regular physical activity, weight management, timely communication with healthcare professionals, and compliance with dietary recommendations. Effective self-care has been associated with improved symptom control, enhanced quality of life, reduced hospitalizations, and lower healthcare costs. Nevertheless, many patients experience difficulties in maintaining adequate self-care behaviors, resulting in suboptimal disease management and poorer clinical outcomes [4].

Dietary management is an important component of HF care. Clinical guidelines emphasize healthy dietary patterns, appropriate sodium intake, fluid management when clinically indicated, and maintenance of adequate nutritional status [4]. Adherence to dietary recommendations may be influenced by multiple individual, social, and cultural factors, making dietary self-management challenging for some patients [6,7,8].

Although both self-care and dietary behaviors are important components of HF management, their relationship has been less extensively investigated, particularly in specific patient populations and healthcare settings. Examining whether dietary habits are associated with broader self-care behaviors may contribute to a better understanding of how these dimensions of HF management coexist in clinical practice. Therefore, the present study aimed to assess self-care behaviors and dietary habits among patients with HF and to examine their associations with each other and with selected demographic and clinical characteristics.

## 2. Materials and Methods

### 2.1. Study Design

A cross-sectional observational study was conducted to assess self-care behaviors and dietary habits and to examine their associations with selected demographic and clinical characteristics among patients with heart failure (HF).

### 2.2. Participants

The study population consisted of patients with HF attending outpatient clinics at a general hospital in Athens, Greece. A total of 137 eligible patients were enrolled between January and June 2026 using convenience sampling.

#### Inclusion and Exclusion Criteria

Patients were eligible for participation if they were aged ≥18 years, had an established diagnosis of HF, were able to read, write, and understand Greek, and provided written informed consent before participation. Patients were excluded if they had a terminal illness or another documented life-threatening condition expected to substantially affect prognosis or their ability to participate; had experienced an acute myocardial infarction or undergone cardiac surgery within the preceding six months; or had a documented history of severe psychiatric illness or cognitive impairment that could interfere with study participation or the reliable completion of the questionnaires. Cognitive impairment and severe psychiatric illness were identified based on diagnoses documented in the patients’ medical records; no formal cognitive or psychiatric screening instrument was administered specifically to determine study eligibility.

### 2.3. Data Collection Instruments

Data were collected using a demographic and clinical data form and two validated self-report questionnaires assessing self-care behaviors and dietary habits.

#### 2.3.1. Demographic and Clinical Data Form

A structured form was used to collect participants’ demographic and clinical characteristics, including age, sex, marital status, educational level, employment status, and NYHA functional class.

#### 2.3.2. Hippocratic Heart Failure Self-Care Scale (HHFSCS)

Patients’ self-care behaviors were assessed using the Hippocratic Heart Failure Self-Care Scale (HHFSCS), a validated instrument for assessing self-care behaviors in patients with heart failure [9]. The HHFSCS consists of 22 items covering medication adherence (items 1–4), nutrition (items 5–10), physical activity (item 11), alcohol consumption (items 13–14), smoking behavior (item 15), symptom monitoring (items 12 and 16–18), attendance at scheduled healthcare appointments and laboratory examinations (items 19–20), and immunization practices (items 21–22). Responses are rated on a five-point Likert scale ranging from “Never” (0) to “Very Often” (4). The total score ranges from 0 to 88, with higher scores indicating better overall self-care. The validity and reliability of the instrument have been previously established [9].

#### 2.3.3. Cardiovascular Diet Questionnaire-2 (CDQ-2)

Dietary habits were assessed using the Cardiovascular Diet Questionnaire-2 (CDQ-2), a validated 17-item instrument designed to assess dietary habits relevant to cardiovascular health [10]. The questionnaire assesses four dietary components: saturated fatty acids (SFA; nine items), monounsaturated fatty acids (MUFA; one item), omega-3 fatty acids (ω3FA; three items), and fruit and vegetable consumption (FV; four items). Component scores range from 0–27 for SFA, 0–6 for MUFA, 0–10 for ω3FA, and 0–14 for FV. The overall dietary score is calculated as (FV + MUFA + ω3FA) − SFA, with higher scores indicating better overall dietary quality; no universally accepted cut-off values have been established [10]. The Greek version of the CDQ-2 has been translated and validated, demonstrating excellent internal consistency (Cronbach’s α = 0.97) [11].

### 2.4. Data Collection Procedure

Data collection was conducted by the research team at the participating outpatient HF clinics. Eligible patients were identified according to the predefined inclusion and exclusion criteria during scheduled clinic visits. After providing written informed consent, participants were enrolled in the study.

Participants received standardized instructions and completed the study questionnaires in a quiet environment. The questionnaires were self-administered, with members of the research team available to provide clarification when necessary without influencing participants’ responses.

Upon completion, questionnaires were reviewed for completeness. When appropriate, participants were invited to complete unintentionally omitted items. The completed questionnaires were subsequently coded and prepared for statistical analysis.

### 2.5. Ethical Considerations

The study was conducted in accordance with the General Data Protection Regulation (GDPR; EU Regulation 2016/679), which governs the protection of personal and sensitive data, and adhered to the ethical principles of the Declaration of Helsinki [12]. Ethical approval was obtained from the Ethics Committee of the Faculty of Nursing, National and Kapodistrian University of Athens (Approval No. 40610/07-04-2026), as well as from the Scientific Committee (147054/24-11-2025) of all participating hospitals before data collection.

Before enrollment, all eligible participants received verbal and written information regarding the study and provided written informed consent. Participation was entirely voluntary, and participants were informed of their right to withdraw from the study at any time without any consequences for their medical care.

To ensure confidentiality and anonymity, each participant was assigned a unique identification code that was used throughout the data collection and analysis process. The correspondence between participants’ identities and their identification codes was securely maintained and was accessible only to the principal investigator.

### 2.6. Statistics

Statistical analyses were performed using IBM SPSS Statistics version 30.0 (IBM Corp., Armonk, NY, USA). Continuous variables are presented as mean ± standard deviation (SD) and categorical variables as frequencies and percentages.

A power analysis for multiple linear regression was performed using G*Power version 3.1. Assuming a medium effect size (f^2^ = 0.15), a two-sided α level of 0.05, and 80% power, the estimated minimum sample size was 118 participants for a model with 10 predictors and 127 participants for a model with 12 predictors. The final sample of 137 participants exceeded these estimated minimum requirements.

The distribution of continuous variables was assessed using the Kolmogorov–Smirnov and Shapiro–Wilk tests. Because the total HHFSCS score and its subscale scores significantly deviated from normality (all *p* < 0.001), non-parametric methods were used for univariable analyses. Associations between continuous variables were examined using Spearman’s rank correlation coefficient. Differences between two independent groups were assessed using the Mann–Whitney U test, whereas comparisons among three or more independent groups were performed using the Kruskal–Wallis test.

To account for the increased risk of type I errors arising from multiple comparisons, the Holm–Bonferroni procedure was applied within prespecified families of conceptually related tests. For analyses of demographic characteristics, an adjustment was performed separately for each demographic factor across the HHFSCS outcomes. For correlations between dietary measures and self-care, an adjustment was performed separately for each CDQ-2 measure across the nine HHFSCS outcomes. Holm-adjusted *p* values < 0.05 were considered statistically significant.

Two multivariable general linear models (GLMs) were performed with the total HHFSCS score as the dependent variable to examine adjusted associations between dietary measures and self-care. Both models were adjusted for age, sex, marital status, educational level, and employment status. In the first model, overall dietary quality, assessed using the total CDQ-2 score, was included as a covariate. In the second model, the total CDQ-2 score was replaced by the individual dietary component scores for saturated fatty acids (SFA), fruit and vegetables (FV), and monounsaturated fatty acids (MUFA). Sex, marital status, educational level, and employment status were entered as fixed factors, whereas age and the dietary variables were entered as covariates. Model fit was summarized using R^2^ and adjusted R^2^, and effect sizes for model terms were expressed as partial eta squared (η^2^).

Unless otherwise specified for the Holm–Bonferroni-adjusted analyses, all statistical tests were two-tailed, and *p* < 0.05 was considered statistically significant.

## 3. Results

### 3.1. Demographic Characteristics of the Study Population

A total of 137 patients were included, with a mean age of 69.3 ± 11.3 years; 54.0% were male. Most participants were retired, and approximately one-third had completed higher education. Among participants with available NYHA functional class data (*n* = 103), 28.2% were classified as NYHA class II and 71.8% as NYHA classes III–IV (Table 1).

### 3.2. Self-Care Behavior

Overall self-care was moderate, with higher scores observed for medication adherence and appointment keeping and lower scores for exercise-, smoking-, alcohol-, and symptom-related self-care behaviors (Table 2).

After Holm–Bonferroni adjustment for multiple comparisons, age, marital status, educational level, and employment status showed significant associations with selected HHFSCS outcomes, whereas no significant differences were observed according to sex (Table 3). Employment status showed the most consistent associations across self-care domains. Total HHFSCS scores did not differ significantly between participants with NYHA class II (median = 47.0, IQR = 8.0) and those with NYHA classes III–IV (median = 45.0, IQR = 12.0; Mann–Whitney U = 1002.0, Z = −0.522, *p* = 0.602).

### 3.3. CDQ-2

The mean total CDQ-2 score was 8.57 ± 7.17 (range: −6 to 28). Descriptive statistics for the individual dietary components are presented in Table 4.

No statistically significant associations were observed between demographic characteristics and CDQ-2 component or total scores, and all findings remained non-significant after Holm–Bonferroni adjustment for multiple comparisons (Supplementary Table S1).

### 3.4. CDQ-2 and HHFSCS

No statistically significant correlation was observed between the total HHFSCS score and either the CDQ-2 component scores or the total CDQ-2 score. Among the HHFSCS subscales, only the correlation between the diet subscale and SFA remained statistically significant after Holm–Bonferroni adjustment (ρ = 0.265, unadjusted *p* = 0.002; adjusted *p* = 0.018). Other initially observed associations did not remain significant after adjustment for multiple comparisons (Supplementary Table S2).

In the multivariable GLM adjusted for age, sex, marital status, educational level, and employment status, the overall model was statistically significant (F(10,125) = 5.701, *p* < 0.001; R^2^ = 0.313; adjusted R^2^ = 0.258). However, the total CDQ-2 score was not significantly associated with the total HHFSCS score after adjustment (B = −0.102, 95% CI [−0.338, 0.134], *p* = 0.394). Employment status remained significantly associated with the total HHFSCS score (overall *p* < 0.001), whereas age, sex, marital status, and educational level were not statistically significant (Table 5).

In the multivariable general linear model, the overall model was statistically significant (F(12,123) = 5.356, *p* < 0.001), explaining 34.3% of the variance in total HHFSCS score (R^2^ = 0.343; adjusted R^2^ = 0.279). Among the dietary components, only the SFA score was significantly associated with total self-care (B = 0.561, 95% CI: 0.111 to 1.011, *p* = 0.015), indicating that each one-unit increase in SFA score was associated with a 0.561-point-higher total HHFSCS score after adjustment for the other variables included in the model. FV (B = 0.235, 95% CI: −0.571 to 1.040, *p* = 0.566) and MUFA scores (B = 0.063, 95% CI: −0.272 to 0.399, *p* = 0.709) were not significantly associated with total self-care (Table 6).

## 4. Discussion

The present study examined the association between self-care behaviors and dietary habits among patients with HF using two validated instruments, HHFSCS and the CDQ-2. Overall dietary quality was not significantly associated with total self-care in either the univariable or adjusted analyses. However, an association was observed between the SFA component and total self-care after adjustment for demographic characteristics. Self-care performance also varied across individual HHFSCS domains, and employment status was the only demographic factor that remained significantly associated with total HHFSCS score in the adjusted models.

Analysis of the individual HHFSCS domains showed higher scores for medication adherence and attendance at scheduled medical appointments and lower scores for exercise, smoking-related behaviors, alcohol-related behaviors, and symptom monitoring. Similar variation across self-care behaviors has been reported in previous studies of patients with HF [13,14,15,16,17]. Lifestyle-related behaviors, including healthy eating and regular physical activity, may be more difficult to maintain than routine self-care behaviors [18,19], supporting the importance of comprehensive self-care approaches that address multiple behavioral domains rather than focusing solely on medication adherence [20,21].

Demographic characteristics have shown inconsistent associations with self-care in previous studies [22,23]. In the present study, several demographic characteristics were associated with selected self-care outcomes in the univariable analyses; however, employment status was the only demographic factor that remained significantly associated with total HHFSCS score after adjustment for the other variables included in the models. Age, sex, marital status, and educational level were not significantly associated with total self-care in the adjusted analysis. Furthermore, no significant difference in total HHFSCS score was observed between participants with NYHA class II and those with NYHA classes III–IV among participants with available NYHA data. The association with employment status should be interpreted cautiously, as employment may reflect broader socioeconomic and social circumstances that were not directly assessed in the present study.

Contrary to our initial hypothesis, overall dietary quality, as assessed by the total CDQ-2 score, was not significantly associated with total self-care. No significant correlation was observed between total CDQ-2 and total HHFSCS scores in the univariable analysis, and the total CDQ-2 score remained non-significant after adjustment for age, sex, marital status, educational level, and employment status. Thus, the present findings do not support an association between overall dietary quality and overall self-care in this sample.

When the individual dietary components were examined separately, SFA was significantly associated with total HHFSCS score after adjustment for demographic characteristics, whereas FV and MUFA were not. This component-specific finding should be interpreted cautiously, particularly given the cross-sectional design and the absence of a significant association between overall dietary quality and total self-care. The mechanisms underlying the observed association with SFA cannot be determined from the present data, as potentially relevant factors such as dietary knowledge, self-efficacy, motivation, and food-related decision-making were not assessed. Accordingly, this finding should be considered exploratory and requires confirmation in other HF populations using more comprehensive dietary and behavioral assessments [24].

Although SFA intake is an important component of dietary quality, the isolated association observed in this study should not be interpreted as evidence that SFA intake influences self-care. Likewise, the absence of significant associations for FV and MUFA does not establish a lack of clinical relevance for these dietary components. Current cardiovascular recommendations emphasize overall dietary patterns rather than individual nutrients [25,26], and the present cross-sectional findings do not permit conclusions regarding the effects of specific dietary components on self-care or cardiovascular outcomes.

Studies using other HF-specific patient-reported measures provide complementary evidence regarding the multidimensional nature of HF management. Previous research has demonstrated variation in adherence across individual self-care recommendations, including medication use, exercise, diet, weight monitoring, and symptom management [27]. In the SODIUM-HF trial, dietary sodium reduction did not significantly reduce major clinical events but was associated with a modest improvement in KCCQ-assessed quality of life [28,29]. These findings highlight that dietary behaviors represent only one component of the broader management of HF.

Dietary assessment and counseling nevertheless remain established components of comprehensive HF management, irrespective of the absence of an association between overall CDQ-2 and self-care in the present study. Current international guidelines support nutritional assessment and individualized dietary counseling as part of multidisciplinary HF care [30,31]. From a nursing perspective, cardiovascular nurses can contribute to the assessment of self-care and dietary behaviors, identify barriers to recommended behaviors, and provide individualized education within nurse-led HF programs. However, the present study does not establish that dietary counseling or nutritional assessment improves self-care or clinical outcomes, and prospective intervention studies are required to evaluate these potential effects.

The present study provides data from a Greek HF population on the relationship between multidimensional self-care behaviors and dietary quality using validated assessment tools. The simultaneous assessment of overall and domain-specific self-care, dietary characteristics, and demographic factors allowed these relationships to be examined within the same population. In addition, multivariable analyses enabled an evaluation of dietary and demographic factors after adjustment for other variables included in the models.

Several limitations should be considered when interpreting the findings. First, the cross-sectional design precludes conclusions regarding temporal direction or causality. Dietary habits were assessed using the CDQ-2 without complementary dietary recalls or a comprehensive food-frequency questionnaire. Although the CDQ-2 is a validated screening instrument, the absence of more detailed dietary assessment limited the characterization of specific food and nutrient intakes and broader dietary patterns.

Second, both self-care and dietary habits were assessed using self-reported questionnaires. Although the HHFSCS and CDQ-2 are validated instruments, self-reported measures are susceptible to recall and social desirability bias and may result in over- or under-reporting of behaviors. Furthermore, because both the outcome and dietary variables were reported by the same participants using questionnaires, common-method bias cannot be excluded. Consequently, observed associations between specific dietary and self-care measures may partly reflect shared measurement characteristics rather than underlying behavioral relationships. No objective or complementary measures of self-care or dietary intake were available to corroborate the questionnaire-based findings.

Third, several potentially important clinical and social factors were not comprehensively assessed or included in the multivariable models, including disease severity, cognitive function, depressive symptoms, health literacy, socioeconomic status, social support, and access to healthcare. Residual confounding by these and other unmeasured factors therefore cannot be excluded. In addition, NYHA functional class was unavailable for 24.3% of participants, and analyses involving functional status were consequently restricted to participants with available data.

Fourth, participants were recruited using convenience sampling from outpatient HF clinics at a single hospital, which may limit the generalizability of the findings to patients treated in other healthcare settings and populations.

Future longitudinal and interventional studies are needed to further examine the relationship between dietary behaviors and self-care in HF individuals and to establish its temporal direction and potential clinical relevance. Such studies should incorporate more comprehensive and objective dietary assessments together with relevant clinical and psychosocial factors, including disease severity, health literacy, cognitive function, psychological well-being, self-efficacy, social support, and socioeconomic circumstances. Multicenter studies involving larger and more diverse populations would also help determine the reproducibility and generalizability of the present findings.

## 5. Conclusions

The present study examined the relationship between dietary characteristics and self-care behaviors among patients with HF. Overall dietary quality, as assessed by the total CDQ-2 score, was not significantly associated with total self-care after adjustment for demographic characteristics. Among the individual dietary components, SFA was significantly associated with total HHFSCS score, whereas FV and MUFA were not. Given the cross-sectional design and reliance on self-reported measures, this component-specific finding should be interpreted cautiously and requires confirmation in prospective studies.

Dietary assessment and counseling remain important components of comprehensive HF management. Future longitudinal and interventional studies incorporating more detailed dietary assessments, objective measures, and relevant clinical and psychosocial factors are needed to clarify the relationship between dietary behaviors and self-care and to determine its potential clinical relevance.

## Figures and Tables

**Table 1:** Baseline demographic characteristics of the study participants (N = 137).

Variable	Value
Age (years), mean ± SD	69.3 ± 11.2
Age range (years)	30–93
Male sex, n (%)	74 (54.0)
Female sex, n (%)	63 (46.0)
Marital status, n (%)	
Married	55 (40.1)
Divorced/Widowed	71 (51.8)
Single	11 (8.0)
Educational level, n (%)	
Compulsory education	50 (36.5)
Secondary education	44 (32.1)
Higher/University education	43 (31.4)
Employment status, n (%)	
Employed	22 (16.1)
Unemployed	40 (29.2)
Retired	75 (54.7)

**Table 2:** Descriptive statistics of the HHFSCS total score and its subscales.

Variable	Mean ± SD	Minimum	Maximum
Total HHFSCS score	41.00 ± 11.27	14	59
Medication	7.91 ± 4.37	0	15
Diet	10.21 ± 3.19	4	16
Exercise	2.91 ± 1.26	0	4
Alcohol	2.89 ± 2.33	0	7
Smoking	1.65 ± 1.62	0	4
Symptom management	5.83 ± 2.50	1	12
Appointment keeping	4.17 ± 3.03	0	8
Vaccination	5.42 ± 2.67	0	8

**Table 3:** Association between demographic characteristics and HHFSCS total score and subscale scores.

Variable	Statistical Test	Total HHFSCS	Medication	Diet	Exercise	Alcohol	Smoking	Symptoms	Appointment	Vaccination
**Age**	Spearman’s ρ (Holm-adjusted p)	−0.250 (0.018)	−0.271 (0.009) *	0.038 (0.659)	−0.303 (<0.01) *	−0.181 (0.105)	−0.244 (0.020)	0.173 (0.105)	−0.297 (<0.01) *	−0.233 (0.024)
**Sex**	Mann–Whitney (Holm-adjusted p)	1.000	1.000	1.000	1.000	0.801	1.000	1.000	1.000	1.000
**Marital status**	Kruskal–Wallis (Holm-adjusted p)	<0.01 *	<0.01 *	0.300	<0.01 *	<0.01 *	0.040	0.042	<0.01 *	0.074
**Educational level**	Kruskal–Wallis (Holm-adjusted p)	0.156	0.154	0.384	0.040	0.256	0.185	0.384	0.036	0.354
**Employment status**	Kruskal–Wallis (Holm-adjusted p)	<0.01 *	<0.01 *	0.527	<0.01 *	<0.01 *	<0.01 *	0.478	<0.01 *	0.060

* For comparisons reported by SPSS as *p* < 0.001, the exact Holm-adjusted *p*-value could not be calculated from the rounded output; these associations remained statistically significant after adjustment.

**Table 4:** Descriptive statistics of the CDQ-2 scores.

Variable	Mean ± SD	Min	Max
SFA	5.99 ± 3.86	1	16
FV	5.68 ± 2.24	1	12
MUFA	8.88 ± 5.16	1	20
Total CDQ-2 score	8.57 ± 7.17	−6	28

CDQ-2, Cardiovascular Diet Questionnaire-2; MUFA, monounsaturated fatty acids; SFA, saturated fatty acids; FV, fruit and vegetables.

**Table 5:** Multivariable general linear model examining adjusted associations with the total HHFSCS score.

Variable	B	95% CI	*p*-Value
Total CDQ-2 score	−0.102	−0.338 to 0.134	0.394
Age (years)	−0.115	−0.281 to 0.051	0.173
Sex (Male vs. Female)	2.505	−1.073 to 6.083	0.168
Marital status	—	—	0.353
Educational level	—	—	0.217
Employment status	—	—	<0.001

Model statistics: *F*(10,125) = 5.701, *p* < 0.001; R^2^ = 0.313; adjusted R^2^ = 0.258. Note: For categorical predictors with more than two levels (marital status, educational level, and employment status), *p*-values represent the overall effect of the variable in the model. Abbreviations: HHFSCS, Hippocratic Heart Failure Self-Care Scale; CDQ-2, Cardiovascular Diet Questionnaire-2; CI, confidence interval.

**Table 6:** Multivariable general linear model examining the association between dietary components and total HHFSCS score.

Variable	B	95% CI	F	df	*p*-Value
Sex	—	—	2.439	1	0.121
Marital status	—	—	0.690	2	0.504
Educational level	—	—	1.890	2	0.155
Employment status	—	—	6.574	3	<0.001
Age	−0.100	−0.265 to 0.065	1.442	1	0.232
SFA score	0.561	0.111 to 1.011	6.102	1	0.015
FV score	0.235	−0.571 to 1.040	0.332	1	0.566
MUFA score	0.063	−0.272 to 0.399	0.140	1	0.709

B coefficients and 95% confidence intervals are presented for continuous predictors. For categorical variables, F-statistics represent omnibus tests across categories. MUFA, monounsaturated fatty acids; SFA, saturated fatty acids; FV, fruit and vegetables.

## Data Availability

The data presented in this study are available on reasonable request from the corresponding authors. The data are not publicly available due to privacy and ethical restrictions related to human participant data.

## Supplementary Materials

The following supporting information can be downloaded at: https://www.mdpi.com/article/10.3390/nu18172745/s1, Table S1. Associations between demographic characteristics and CDQ-2 scores. Table S2. Spearman correlations between HHFSCS and CDQ-2 scores.

## Abbreviations

The following abbreviations are used in this manuscript:

HF	Heart Failure
HHFSCS	Hippocratic Heart Failure Self-Care Scale
CDQ-2	Cardiovascular Diet Questionnaire-2
SFA	Saturated fatty acids
FV	Fruit and vegetables
MUFA	Monounsaturated fatty acids

## References

[1] World Health Organization (2025). Cardiovascular Diseases (CVDs). https://www.who.int/news-room/fact-sheets/detail/cardiovascular-diseases-(cvds).

[2] Heart Failure Society of America (2024). HF Stats 2024: Heart Failure Epidemiology and Outcomes Statistics. https://hfsa.org/hf-stats-2024-heart-failure-epidemiology-and-outcomes-statistics.

[3] Seferović P.M., Vardas P., Jankowska E.A., Maggioni A.P., Timmis A., Milinković I., Polovina M., Gale C.P., Lund L.H., Lopatin Y. (2021). The Heart Failure Association Atlas: Heart Failure Epidemiology and Management Statistics 2019. Eur. J. Heart Fail..

[4] American Heart Association, A (2022). Heart Disease and Stroke Statistics—2022 Update A Report From the American Heart Association. Circulation.

[5] World Health Organization (1983). Health Education in Selfcare: Possibilities and Limitations.

[6] Dos Reis Padilha G., Sanches Machado d’Almeida K., Ronchi Spillere S., Corrêa Souza G. (2018). Dietary Patterns in Secondary Prevention of Heart Failure: A Systematic Review. Nutrients.

[7] Bechthold A., Boeing H., Schwedhelm C., Hoffmann G., Knüppel S., Iqbal K., De Henauw S., Michels N., Devleesschauwer B., Schlesinger S. (2019). Food groups and risk of coronary heart disease, stroke and heart failure: A systematic review and dose-response meta-analysis of prospective studies. Crit. Rev. Food Sci. Nutr..

[8] García-García A., Alvarez-Sala-Walther L.A., Lee H.Y., Sierra C., Pascual-Figal D., Camafort M. (2022). Is there sufficient evidence to justify changes in dietary habits in heart failure patients? A systematic review. Korean J. Intern. Med..

[9] Brokalaki H., Chatziefstratiou A.A., Fotos N.V., Patelarou A., Giakoumidakis K. (2024). The Development and Testing of the Hippocratic Heart Failure Self-Care Scale. Healthcare.

[10] Paillard F., Flageul O., Mahé G., Laviolle B., Dourmap C., Auffret V. (2021). Validation and reproducibility of a short food frequency questionnaire for cardiovascular prevention. Arch. Cardiovasc. Dis..

[11] Giakoumidakis K., Patelarou E., Chatziefstratiou A.A., Aloizou D., Vaitsis N., Brokalaki H., Fotos N.V., Geniataki E., Patelarou A.E. (2025). Validation of the Greek Cardiovascular Diet Questionnaire 2 (CDQ-2) and Single-Center Cross-Sectional Insights into the Dietary Habits of Cardiovascular Patients. Nutrients.

[12] World Medical Association Declaration of Helsinki (2013). Ethical principles for medical research involving human subjects. JAMA.

[13] Negarandeh R., Hoseini-Esfidarjani S.S., Aghajanloo A. (2026). Self-Care Behaviors in Heart Failure Patients Using the European Heart Failure Self-Care Behavior Scale: Systematic Review and Meta-Analysis. Florence Nightingale J. Nurs..

[14] Opasrattanakon S., Jaraeprapal U., Punsawad C. (2026). Self-care management in patients with heart failure in Nakhon Si Thammarat Province, Thailand: A descriptive qualitative study. Sci. Rep..

[15] Kim S.H., Lim A., Benjasirisan C., Himmelfarb C.R., Davidson P.M., Wright R., Koirala B. (2026). Relationships Among Symptom Burden, Self-Care, and Quality of Life Among Individuals Living With Heart Failure and Multimorbidity: A Cross-Sectional Study. J. Clin. Nurs..

[16] Riegel B., Moser D.K., Anker S.D., Appel L.J., Dunbar S.B., Grady K.L., Gurvitz M.Z., Havranek E.P., Lee C.S., Lindenfeld J. (2009). State of the science: Promoting self-care in persons with heart failure: A scientific statement from the American Heart Association. Circulation.

[17] Jaarsma T., Hill L., Bayes-Genis A., La Rocca H.B., Castiello T., Čelutkienė J., Marques-Sule E., Plymen C.M., Piper S.E., Riegel B. (2021). Self-care of heart failure patients: Practical management recommendations from the Heart Failure Association of the European Society of Cardiology. Eur. J. Heart Fail..

[18] Riegel B., Jaarsma T., Strömberg A. (2012). A middle-range theory of self-care of chronic illness. ANS Adv. Nurs. Sci..

[19] Riegel B., Jaarsma T., Lee C.S., Strömberg A. (2019). Integrating Symptoms Into the Middle-Range Theory of Self-Care of Chronic Illness. ANS Adv. Nurs. Sci..

[20] Narimisa F., Karbakhsh S., Pouradeli S. (2026). Telenursing and Self-Efficacy in Heart Failure. Crit. Care Nurs. Q..

[21] Sallam L.A., AlOtaibi N.G., Baker O.G. (2026). Effectiveness of Telehealth Care on Self-care Behaviours in Heart Failure Patients: Systematic Review and Meta-Analysis. SAGE Open Nurs..

[22] Ausili D., Rebora P., Di Mauro S., Riegel B., Valsecchi M.G., Paturzo M., Alvaro R., Vellone E. (2016). Clinical and socio-demographic determinants of self-care behaviours in patients with heart failure and diabetes mellitus: A multicentre cross-sectional study. Int. J. Nurs. Stud..

[23] Tian F., Chen S., Lan X., Zhang R.X., Zhu C.Y., Chen Y. (2026). Factors influencing self-care behaviours in patients with heart failure: A mixed-methods systematic review. Heart Lung J. Crit. Care.

[24] Riegel B., Jaarsma T., Strömberg A. (2026). An Update to the Middle-Range Theory of Self-Care of Chronic Illness. ANS. Adv. Nurs. Sci..

[25] Visseren F.L.J., Mach F., Smulders Y.M., Carballo D., Koskinas K.C., Bäck M., Benetos A., Biffi A., Boavida J.M., Capodanno D. (2021). 2021 ESC Guidelines on cardiovascular disease prevention in clinical practice. Eur. Heart J..

[26] McDonagh T.A., Metra M., Adamo M., Gardner R.S., Baumbach A., Böhm M., Burri H., Butler J., Čelutkienė J., Chioncel O. (2023). 2023 Focused Update of the 2021 ESC Guidelines for the diagnosis and treatment of acute and chronic heart failure. Eur. Heart J..

[27] Marti C.N., Georgiopoulou V.V., Giamouzis G., Cole R.T., Deka A., Tang W.H.W., Dunbar S.B., Smith A.L., Kalogeropoulos A.P., Butler J. (2013). Patient-reported selective adherence to heart failure self-care recommendations: A prospective cohort study: The Atlanta Cardiomyopathy Consortium. Congest. Heart Fail..

[28] Green C.P., Porter C.B., Bresnahan D.R., Spertus J.A. (2000). Development and evaluation of the Kansas City Cardiomyopathy Questionnaire: A new health status measure for heart failure. J. Am. Coll. Cardiol..

[29] Spertus J.A., Jones P.G., Sandhu A.T., Arnold S.V. (2020). Interpreting the Kansas City Cardiomyopathy Questionnaire in Clinical Trials and Clinical Care: JACC State-of-the-Art Review. J. Am. Coll. Cardiol..

[30] McDonagh T.A., Metra M., Adamo M., Gardner R.S., Baumbach A., Böhm M., Burri H., Butler J., Čelutkienė J., Chioncel O. (2021). 2021 ESC Guidelines for the diagnosis and treatment of acute and chronic heart failure. Eur. Heart J..

[31] Heidenreich P.A., Bozkurt B., Aguilar D., Allen L.A., Byun J.J., Colvin M.M., Deswal A., Drazner M.H., Dunlay S.M., Evers L.R. (2022). 2022 AHA/ACC/HFSA Guideline for the Management of Heart Failure: A Report of the American College of Cardiology/American Heart Association Joint Committee on Clinical Practice Guidelines. Circulation.

