# Supplementary material for: Self-Care Behaviors and Dietary Habits Among Patients with Heart Failure: A Cross-Sectional Study
Source: Nutrients. 2026 Aug 22;18(17):2745. doi: 10.3390/nu18172745 (PMC13567115; doi:10.3390/nu18172745)
Supplement: Supplementary file 1 [file nutrients-18-02745-s001.zip › nutrients-4447575-supplementary.pdf]

Table S1. Associations between demographic characteristics and CDQ-2 scores.

Variable	Statistical test	SFA	FV	MUFA	Total CDQ-2
Age	Spearman's ρ	-0.107 (0.217)	-0.018 (0.839)	0.121 (0.162)	-0.053 (0.538)
Sex	Mann–Whitney (p)	0.314	0.528	0.090	0.839
Marital status	Kruskal–Wallis (p)	0.942	0.926	0.462	0.780
Educational level	Kruskal–Wallis (p)	0.726	0.601	0.265	0.425
Employment status	Kruskal–Wallis (p)	0.599	0.636	0.540	0.736

CDQ-2, Cardiovascular Diet Questionnaire-2; MUFA, monounsaturated fatty acids; SFA, saturated fatty acids; FV, fruit and vegetables. Statistical significance was evaluated after adjustment for multiple comparisons using the Holm–Bonferroni procedure within each family of related tests.

Table S2. Spearman correlations between HHFSCS and CDQ-2 scores.

HHFSCS variable	SFA ρ (p)	FV ρ (p)	MUFA ρ (p)	Corrected Total CDQ-2 ρ (p)
Total HHFSCS score	0.113 (0.189)	0.002 (0.979)	−0.018 (0.835)	**−0.081 (0.350)**
Medication	0.074 (0.389)	0.020 (0.814)	−0.005 (0.956)	**−0.031 (0.720)**
Diet	**0.265 (0.002)†**	−0.015 (0.867)	0.224 (0.009)	**0.007 (0.936)**
Exercise	−0.002 (0.983)	−0.057 (0.507)	−0.156 (0.070)	**−0.136 (0.113)**
Alcohol	0.007 (0.934)	0.018 (0.838)	−0.085 (0.326)	**−0.066 (0.448)**
Smoking	0.025 (0.770)	0.045 (0.602)	−0.076 (0.378)	**−0.069 (0.425)**
Symptoms	0.003 (0.977)	0.060 (0.486)	0.080 (0.354)	**0.063 (0.467)**
Appointment keeping	0.087 (0.313)	−0.010 (0.906)	−0.064 (0.456)	**−0.094 (0.276)**
Vaccination	0.000 (1.000)	−0.078 (0.365)	−0.125 (0.146)	**−0.119 (0.167)**

Values are presented as Spearman's ϱ (unadjusted p-value). †Statistically significant after Holm–Bonferroni adjustment for multiple comparisons within each set of nine HHFSCS outcomes (adjusted $p < 0.05$). HHFSCS, Heart Failure Self-Care Scale; CDQ-2, Cardiovascular Diet Questionnaire-2; MUFA, monounsaturated fatty acids; SFA, saturated fatty acids; FV, fruit and vegetables.
